# Microcontinent subduction and S-type volcanism prior to India–Asia collision

**DOI:** 10.1038/s41598-021-94492-y

**Published:** 2021-07-21

**Authors:** Zongyao Yang, Juxing Tang, M. Santosh, Xiaoyan Zhao, Xinghai Lang, Ying Wang, Shuai Ding, Fengqin Ran

**Affiliations:** 1 Faculty of Geosciences and Environmental Engineering, Southwest Jiaotong University, Chengdu, 611756 China; 2 Institute of Mineral Resources, Chinese Academy of Geological Sciences, Beijing, 100037 China; 3 School of Earth Sciences and Resources, China University of Geosciences, Beijing, 100083 China; 4 Department of Earth Sciences, University of Adelaide, Adelaide, SA 5005 Australia; 5 College of Earth Science, Chengdu University of Technology, Chengdu, 610059 China

**Keywords:** Geochemistry, Geodynamics, Tectonics

## Abstract

Continental crust has long been considered too buoyant to be subducted beneath another continent, although geophysical evidence in collision zones predict continental crust subduction. This is particularly significant where upper continental crust is detached allowing the lower continental crust to subduct, albeit the mechanism of such subduction and recycling of the upper continental crust remain poorly understood. Here, we investigate Paleocene S-type magmatic and volcanic rocks from the Linzizong volcanic succession in the southern Lhasa block of Tibet. These rocks exhibit highly enriched ^87^Sr/^86^Sr, ^207^Pb/^206^Pb and ^208^Pb/^206^Pb together with depleted ^143^Nd/^144^Nd isotope ratios. The geochemical and isotopic features of these rocks are consistent with those of modern upper continental crust. We conclude that these Paleocene S-type volcanic and magmatic rocks originated from the melting of the upper continental crust from microcontinent subduction during the late stage of India–Asia convergence.

## Introduction

**Figure 1:**
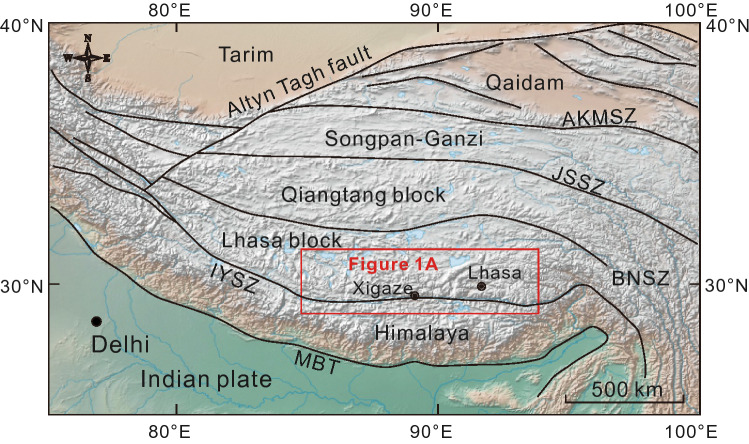
Tectonic and geomorphic map of the Himalayan–Tibetan orogen showing major tectonic units. Base map is made with Natural Earth (https://www.naturalearthdata.com). The map was modified by Zongyao Yang, using CorelDRAW X8 version 18.0.0.448. *AKMSZ* Anyimaqen–Kunlun–Muztagh suture zone, *JSSZ* Jinsha suture zone, *BNSZ* Bangong–Nujiang suture zone, *IYSZ* Indus–Yarlung suture zone, *MBT* Main boundary thrust.

**Figure 2:**
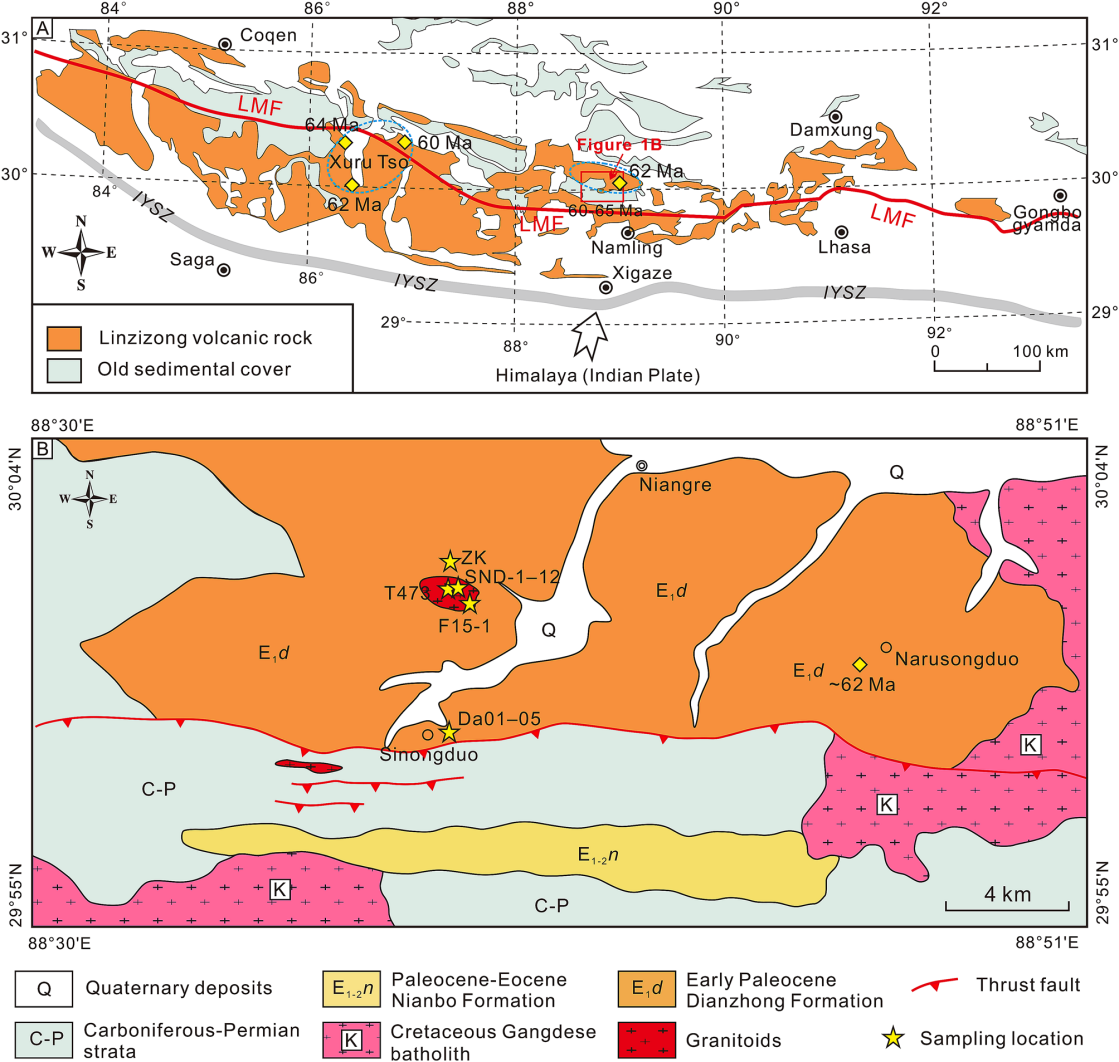
(**A**) Simplified tectonic framework of the southern margin of the Lhasa block showing the location of the study area. (**B**) Regional geological map showing the study area, modified from the China Geological Survey (www.cgs.gov.cn) based on our field observations. The maps were generated by Zongyao Yang, using CorelDRAW X8 version 18.0.0.448. Yellow diamonds represent literature data of Paleocene S-type magma^9–12^. Blue dashed circles represent the approximate extent of the s-type granitic rocks. *LMF* Luobadui–Milashan Fault.

The amalgamation of the Indian and Asian lithospheric plates and the construction of the Himalayan–Tibetan orogens mark one of the most prominent collisional events on the globe^1,2^. The rise of the Qinghai–Tibet Plateau as the highest plateau in the world with an average elevation of around 3000 m is a remarkable outcome of the India–Asia collision along the Indus–Yarlung suture zone (IYSZ)^3^ (Fig. 1). The Linzizong volcanic succession (LVS) and the coeval intrusive rocks in southern Tibet, which cover more than 50% of the Gangdese Belt extending E–W for more than 1200 km (Fig. 2A) are prominent markers of the magmatic activity associated with the India–Asia collision^4–9^.

The LVS has a total thickness of more than 6500 m in the Linzhou Basin and can be subdivided into three groups from the bottom to the top as: the lower Dianzhong, middle Nianbo, and upper Pana Formations with approximate thicknesses of > 3326 m, 845 m, and 2350 m, respectively^4^. Previous geochronological studies defined the eruption boundaries of the Dianzhong, Nianbo, and Pana Formations as 65–60 Ma, 60–54 Ma and ~ 54–43 Ma, respectively^6,7,9^. As evident from their thicknesses, the Dianzhong and Pana Formations indicate more intense volcanic activity than the Nianbo Formation. The Dianzhong and Nianbo Formations show arc-like geochemical signature with significant mantle contributions^5–9,13^, in contrast to the geochemically heterogeneous of the Pana Formation^7,9^. The same inference can also be made for the coeval intrusive rocks as they show similar whole-rock geochemistry and Sr–Nd isotope, and zircon Hf isotope compositions with the Cretaceous I-type Gangdese batholith^6,14–16^. Besides, some hypabyssal granitic rocks show increased crustal contribution similar to S-type granite with radiogenic Sr–Nd–Pb and zircon Hf isotope compositions^10,13,17,18^. In general, the geodynamic setting of the Paleocene Dianzhong volcanic and intrusive rocks was either an Andean-type convergent margin formed by the northward subduction of the Neo-Tethys oceanic crust beneath the Lhasa block^19^, or a syn-collision orogen formed during the rollback of the Neo-Tethys slab accompanied by crustal melting induced by asthenospheric upwelling and magma mixing^8,14–17^. However, our recent studies on the Dianzhong Formation in the southern Lhasa block reveal geochemical characteristics of wholly continental crust-derived melts that are in sharp contrast to the concept of oceanic crust- and mantle source. The origin of these continental crust-derived volcanic and granitic rocks has not been investigated in detail.

In this paper, we report the finding of Paleocene (ca. 65–60 Ma) S-type magmatism in the LVS from the Sinongduo area in southern Tibet. We propose a novel model for the India–Asia collision and the formation of the S-type magma where we envisage of the subduction of microcontinent during the India–Asia convergence, and that the partial melting of the upper continental crust (UCC) of the subducted microcontinent significantly contributed to the formation of the Paleocene S-type volcanic rocks and granitoids.

## Geological background and sampling

The Tibetan Plateau from north to south comprises the Songpan-Ganzi, Qiangtang, Lhasa, and Himalaya blocks (Fig. 1). These continental blocks associated with flysch complexes, and island arcs are considered to have accreted successively to the Asia plate since the early Paleozoic^1^. The Lhasa block, the last block that accreted to the Asia plate before the India–Asia collision, is sandwiched between the Qiangtang block and the Himalayas. Subsequent to this amalgamation and the northward subduction of the Neo-Tethys oceanic crust, the Himalaya (Indian plate) finally collided with Asia in the early Cenozoic along the IYSZ resulting in the uplift of the Tibetan Plateau^2,3,20^.

**Figure 3:**
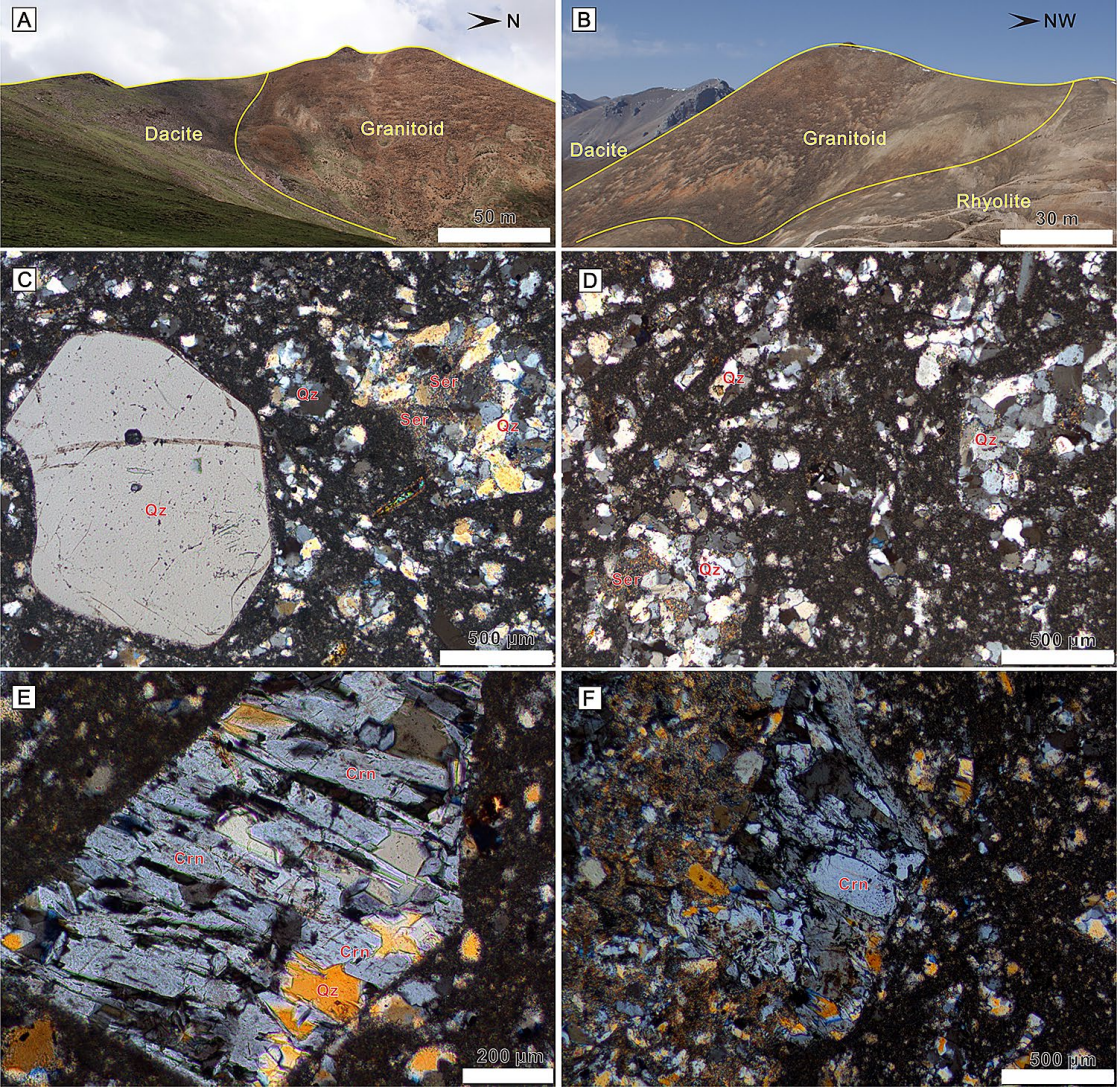
(**A**, **B**) Field photographs showing contact relationships between different types of magmatic rocks. (**C**–**F**) Photomicrographs showing the mineral composition of the granitoids in the Sinongduo area. *Crn* Corundum, *Qz* Quartz, *Ser* Sericite.

The Sinongduo area is located in Xietongmen County, Tibet (Fig. 2A). Widespread volcanic rocks and Carboniferous-Permian strata are exposed in the area (Fig. 2B). The volcanic rocks that consist mainly of rhyolite, tuff, dacite, and volcanic breccia overlying the Cretaceous Gangdese batholith were intruded by a granitic pluton (Fig. 3A,B). Detailed geochronological studies indicate that these volcanic rocks erupted during ~ 65–60 Ma and belong to the Dianzhong Formation of the LVS^21,22^. Therefore, these rocks preserve important information relating to India–Asia collision. In this study, we collected representative samples of both volcanic and intrusive rocks and conduct systematic whole-rock major and trace element, geochronologic, and Sr–Nd–Pb isotope studies with a view to assess their magma sources. Two representative samples (T473 and F15-1) collected from granitoids were used for zircon U–Pb dating. Twelve samples (SND-1 to SND-12) were used for whole-rock major and trace element analyses which were collected from the inner parts of the granitoids. We collected the least-altered and -weathered samples. Microscopic observation indicates that the granitoids are composed mainly of quartz, corundum, and minor orthoclase, muscovite (Fig. 3C–F) with fine-grained porphyritic-like texture. The whole-rock major and trace element data on these volcanic rocks were reported by Ding et al.^21^.

## Geochronology and geochemistry

**Figure 4:**
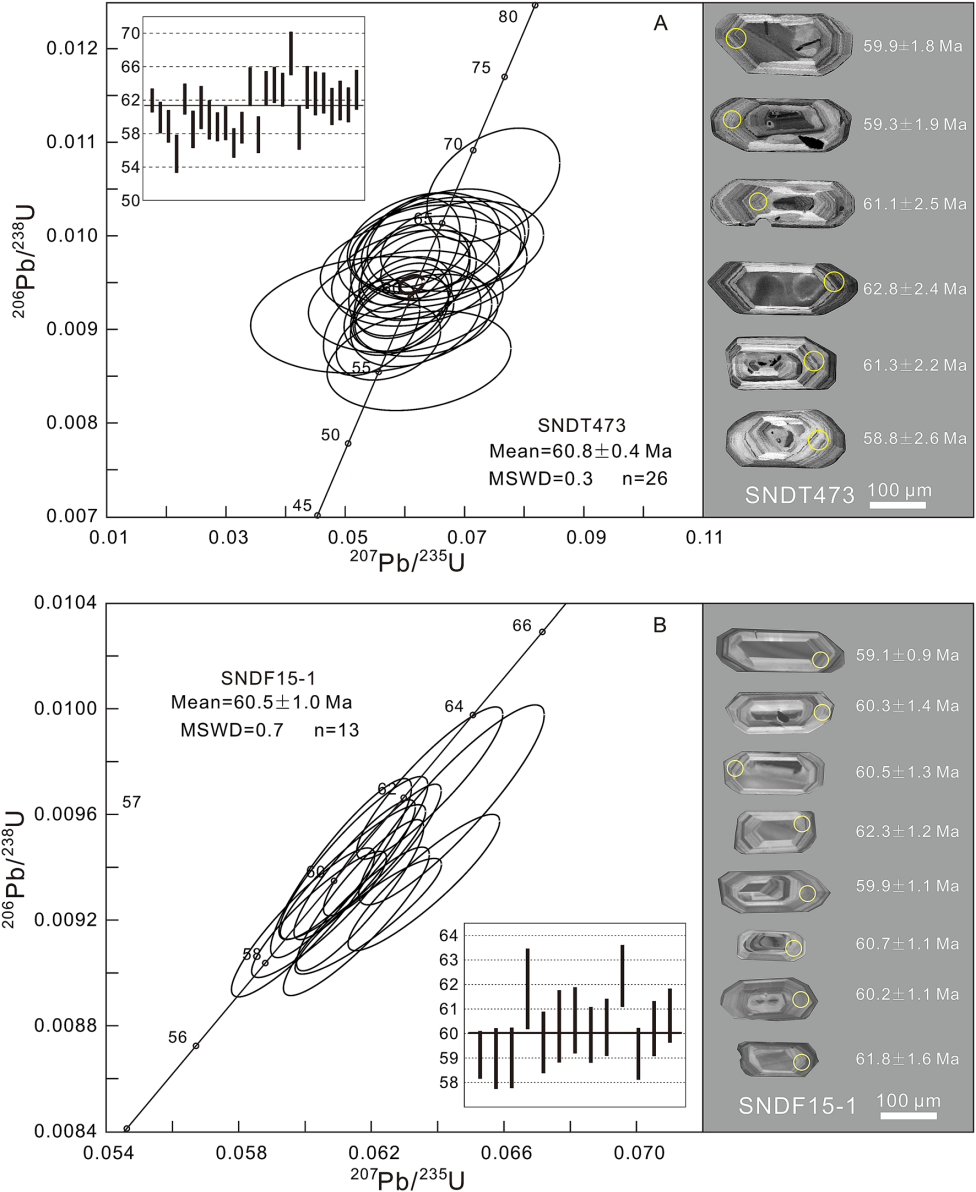
Zircon U–Pb concordia diagram and weighted ages for the Sinongduo Linzizong granitoids T473 (**A**) and F15-1 (**B**) showing typical cathodoluminescence (CL) images of representative zircons with ^238^U/^206^Pb ages.

In the granitoid samples the U–Pb data on 26 zircon grains from sample T473 and 13 from F15-1 yielded concordant ^206^Pb/^238^U ages of 60.8 ± 0.4 Ma (Fig. 4A) (MSWD = 0.3) and 60.5 ± 1.0 Ma (Fig. 4B) (MSWD = 0.7), respectively. Zircons are euhedral and have crystal lengths of ~ 100–250 μm, with length/width ratios from 1:1 to 3:1. The dated zircon grains from samples T473 and F15-1 display oscillatory zoning in cathodoluminescence (CL) images and have variable Th (97–430 ppm and 287–1, 031 ppm, respectively) and U (119–490 ppm and 208–1, 016 ppm, respectively) contents, with Th/U ratios ranging from 0.64 to 1.22 and 0.43 to 1.88, respectively (Supplementary Table S1), suggesting magmatic crystallization. In addition, inherited zircons are not present in both granitoid and volcanic rocks^21^.

**Figure 5:**
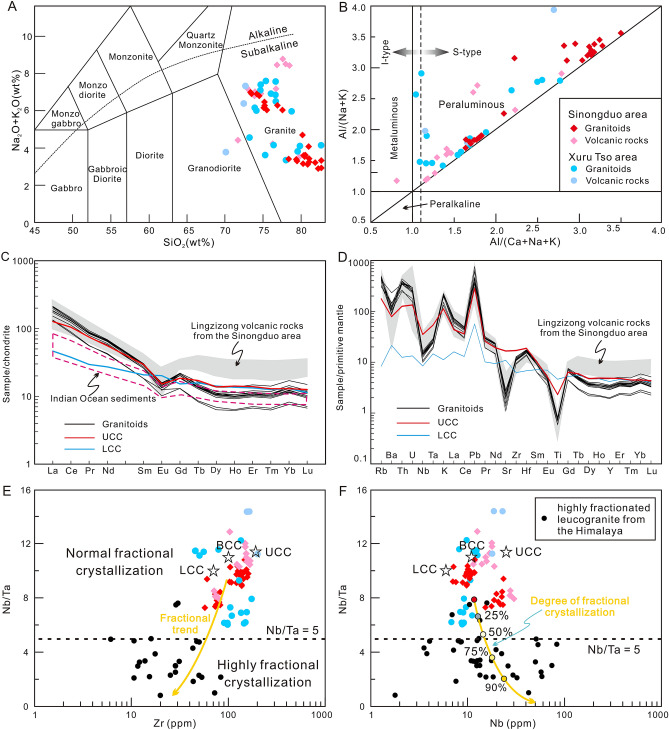
Geochemical characteristics of Paleocene S-type granitic rocks from the southern Lhasa block. (**A**) Total alkali versus silica (TAS) diagram. (**B**) A/NK versus A/CNK classification diagram. (**C**) Chondrite-normalized REE patterns and (**D**) primitive mantle-normalized trace element patterns for Paleocene granitoids from the Sinongduo area. (**E**) Nb/Ta versus Zr and (**F**) Nb/Ta versus Nb diagrams^25^. Chondrite and primitive mantle data are from Sun and McDonough^26^. Data for the UCC, BCC and LCC are from Taylor and McLennan^27^. Data for the Himalayan highly fractionated leucogranite are from Liu et al.^28^, and Lin et al.^29^. Data for the Xuru Tso granitoids and volcanic rocks are from Lee et al.^9^, and Gao et al.^10^. Other data for the Sinongduo granitoids and volcanic rocks are from Yang^11^, Zhou et al.^12^, and Ding et al.^21^.

The chemical compositions of the Linzizong granitoids are listed in Supplementary Table S2. The granitoids from Sinongduo have high SiO_2_ (73.47–81.13 wt%) and K_2_O (4.00–6.77 wt%), moderate Al_2_O_3_ (9.46–14.03 wt%), and uniformly low Na_2_O (0.09–0.18 wt%), CaO (0.14–0.19 wt%)_,_ MgO (0.10–0.23 wt%), and FeO^T^ (0.82–1.40 wt%) contents compared with the normal Dianzhong magmatic rocks^5,6,8,9^. The volcanic rocks and granitoids plot within the rhyolite and granite fields, respectively, on the total alkalis vs. silica (TAS) diagram (Fig. 5A). The alumina saturation index (ASI = mol. Al_2_O_3_/(CaO + Na_2_O + K_2_O)) values of these rocks are in the range of 1.64–2.10, indicating that they are strongly peraluminous (Fig. 5B) and similar to the S-type granites such as those from the Lachlan fold belt^23,24^ (Fig. 5B). As shown in the chondrite-normalized rare earth element (REE) patterns (Fig. 5C), the Linzizong granitoids have enriched light REE (LREE) and relatively flat heavy REE (HREE) patterns with obvious negative Eu anomalies (Eu/Eu* = 0.56–0.82). Their primitive mantle-normalized incompatible trace element patterns (Fig. 5D) exhibit enrichment in some large ion lithophile elements (LILEs) (e.g., Rb, Th, U, and Pb) and depletions in Ba, Sr, and high field strength elements (HFSEs), particularly Nb, Ta, and Ti. The normalized REE and incompatible element abundance curves of the Linzizong granitoids are similar to those of the volcanic rocks from the Sinongduo area, indicating they formed from similar primary magma^21^. Whole-rock Sr–Nd–Pb isotope data for the Linzizong granitoids are listed in Supplementary Table S3. The granitoids have uniform ^87^Sr/^86^Sr ratios of 0.7247–0.7293 and ^143^Nd/^144^Nd ratios of 0.5122–0.5123, whereas the volcanic samples of rhyolite, tuff and dacite exhibit variable ^87^Sr/^86^Sr (0.7146–0.7531) but homogeneous ^143^Nd/^144^Nd (0.5120–0.5122) ratios. The Pb isotope compositions of granitoids are also uniform, with ^206^Pb/^204^Pb, ^207^Pb/^204^Pb, and ^208^Pb/^204^Pb ratios ranging from 18.7522 to 18.7660, 15.7091 to 15.7098, and 39.3622 to 39.4055, respectively.

## Alteration, assimilation and fractional crystallization

Our petrographic observation shows that the rocks underwent low degree sericite alteration (Fig. 3B). The loss-on-ignition (LOI) values of the samples are uniform and vary from 2.04 to 4.18 wt% (average = 2.99 wt%), implying slight alteration. Here, we argue that the geochemical composition can still be employed as a tracer to discuss the magma origin because the content of oxides does not show significant geochemical variation. Besides, the samples are characterized by sub-parallel patterns of REE and trace element concentrations (Fig. 5C,D), indicating they still preserve their original geochemical signatures^30^.

**Figure 6:**
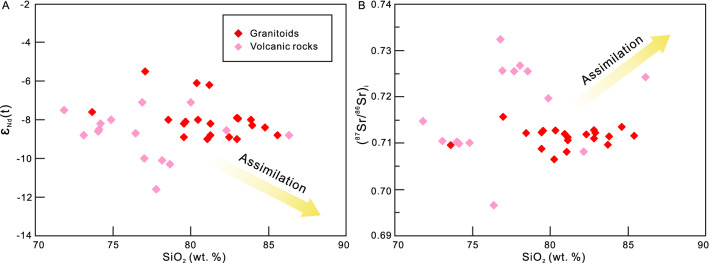
(**A**) SiO_2_ versus ε_Nd_(t) and (**B**) SiO_2_ versus (^87^Sr/^86^Sr)_i_ diagrams. Whole-rock initial ^87^Sr/^86^Sr ratios and ε_Nd_(t) show low correlation with SiO_2_.

Both the granitoids and volcanic rocks have high SiO_2_, which chemically correspond to the high silica granites (HSGs) with SiO_2_ > 70 wt%. The HSGs are considered can be formed by highly fractional crystallization of the mafic parents^31^. The important question is whether the granitoids and volcanic rocks inherited their peraluminous geochemistry from the source region or evolved from a relatively mafic parent^32^ by assimilation and fractional crystallization (AFC). Previous studies consider the Dianzhong volcanic rocks as I-type rocks originated from mantle wedge that containing some crustal component followed by the AFC processes^5–7,9^. However, these S-type volcanic and magmatic rocks have extremely low Na_2_O, CaO_,_ MgO, FeO^T^, Cr (5.20–15.42), Ni (1.09–3.57), and Co (0.60–1.54) contents with radiogenic Sr and Pb isotope composition compared with Dianzhong I-type series^5,6,8,9^, which indicate that they are not likely originated from partial melting of the mantle wedge or the mafic lower continental crust (LCC). Besides, the initial ^87^Sr/^86^Sr ratios and ε_Nd_(t) of the granitoids and volcanic rocks exhibit inconsistent relations rather than any correlation with SiO_2_ contents (Fig. 6A,B), and the volcanic rocks and granitoids also yield relatively uniform Pb isotope compositions (Supplementary Table S3), thus precluding significant crustal assimilation. With respect to the fractional crystallization, we apply high-field-strength elements Nb, Ta and Zr to evaluate the degree of fractional crystallization^25,33^. The Nb and Ta concentrations show a positive correlation with the degree of fractional crystallization, while the Nb/Ta and Zr concentration progressively decrease^33^. The isotope compositions indicate that these magmatic rocks were derived from the upper continental crust. Their Nb/Ta ratios (9.12–10.33) are slightly lower than the upper continental crust (11.36) and different from the highly fractionated Himalaya leucogranites (< 5) (Fig. 5E,F). The low Nb, Ta and high Nb/Ta ratios indicate the granitoids and volcanic rocks from the Sinongduo area could not have experienced strongly fractional crystallization and the geochemistry of these magmatic rocks reflects the composition of the source region.

## Magma source and petrogenesis

**Figure 7:**
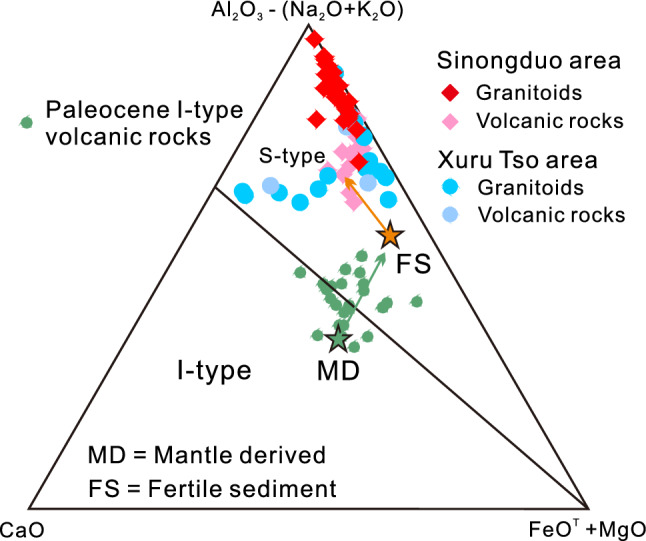
Al_2_O_3_-(Na_2_O + K_2_O) versus CaO versus FeO^T^ + MgO diagram^23^. Data for the Paleocene I-type volcanic rocks are Mo et al.^5,6^, and Lee et al.^9^.

The granitoids and volcanic rocks from the Sinongduo area are strongly peraluminous and plot in the S-type magma field (Figs. 5B, 7), in contrast to the I-type Gangdese batholith and coeval volcanic rocks elsewhere in the Lhasa block. The Lhasa terrane was not under a post-collisional setting in the Paleocene, and therefore these rocks cannot be correlated to post-collisional strongly peraluminous granites formed in such settings^34^. Even though the Sinongduo granitoids and volcanic rocks have elevated Fe-index (0.82–0.92) which is an indicator of the ferroan (A-type) granitoids^35^, the characteristics such as the absence of dark-colored minerals, low 10,000*Ga/Al ratios (1.76–2.58) and Zr + Nb + Ce + Y values (174–257 ppm), indicate that the Sinongduo granitoids are different from the A-type granites^36,37^.

Chappell and White^24^ defined S-type granites as: (1) being strongly peraluminous with ASI > 1.1, (2) containing > 1% CIPW^38^ normative corundum, and (3) having restricted to high SiO_2_ compositions with relatively low Na_2_O (generally < 3.2%) but high K_2_O (~ 5%) contents. As mentioned above, the characteristics of high SiO_2_ and K_2_O, low Na_2_O contents, and ASI values of 1.64–2.10 imply that these granitoids and volcanic rocks have affinity to S-type granite. The normative corundum (Fig. 3E,F) contents of 3.78–6.39% based on the CIPW calculation is even more enriched than the peraluminous felsic S-type series in the Lachlan fold belt and contrasts with the depleted I-type series^24^. The classification of the Sinongduo magmatic rocks as S-type is also supported by their whole-rock initial Sr^87^/Sr^86^ isotopes (0.707–0.732, except ZK0602-2), as Sr is more radiogenic in S-type granites with initial Sr^87^/Sr^86^ > 0.708^24^.

Given that the geochemical characteristics are similar to those of upper crustal sedimentary rocks, S-type magma is considered to form by melting of a sedimentary UCC source that had experienced at least one cycle of weathering^24^ rather than from the more mafic LCC^27^ or oceanic crust. A long-term weathering process can remove Na and Ca and maintain high K in the abundant clay minerals^39^, which leads to the high ASI in sediments. Therefore, the key factor for the generation of S-type granitic magma is a sedimentary source^40^. Since such sediments are deposited only in the Earth’s upper continental crust, S-type granitic magma is considered to be product of anatexis^40,41^.

**Figure 8:**
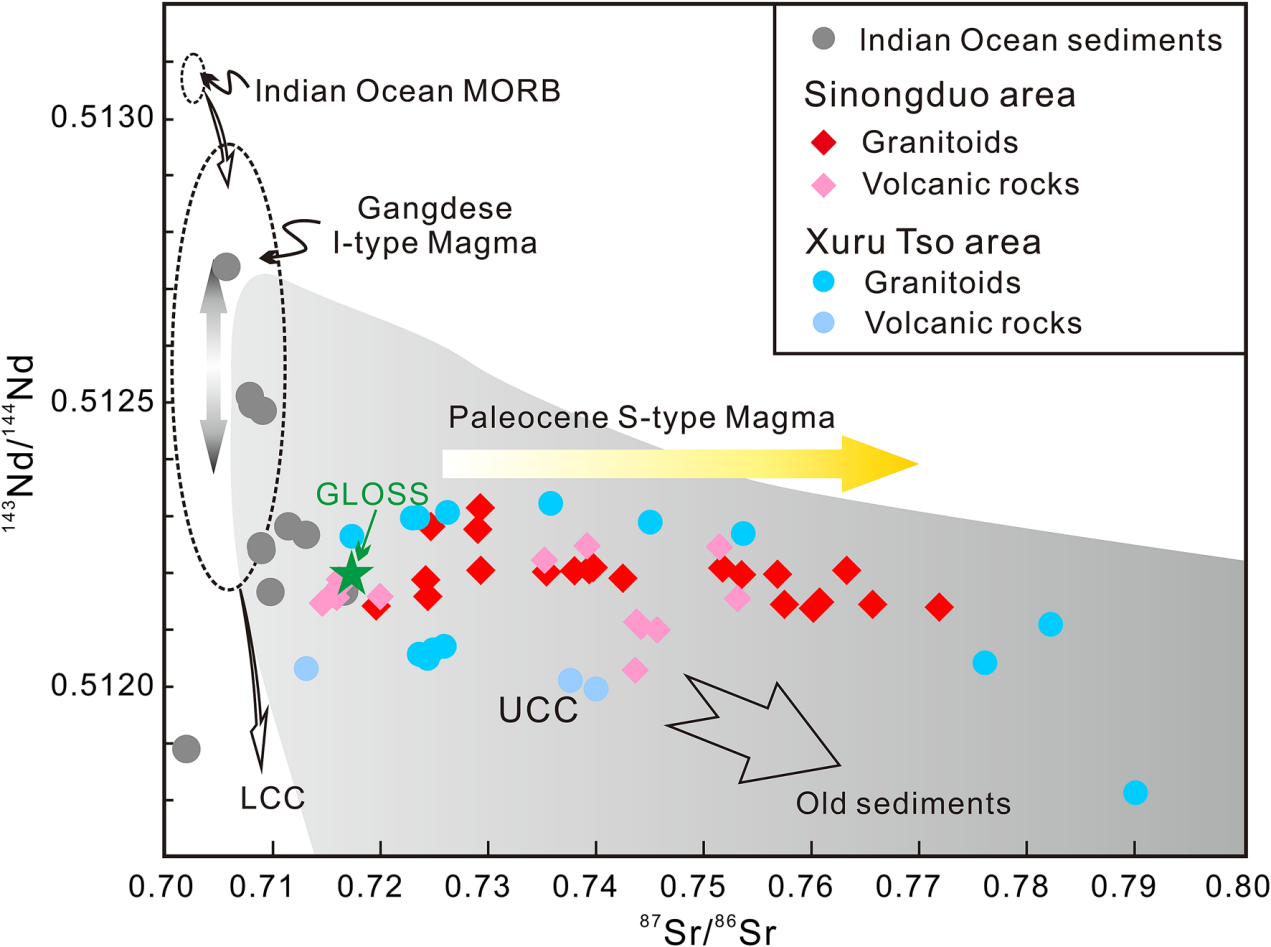
Isotopic evidence for UCC components in Paleocene magma from the Sinongduo area. (A) ^87^Sr/^86^Sr versus ^143^Nd/^144^Nd^42^ for Paleocene magma from the Sinongduo area. Data from the Indian Ocean sediments^43^ are plotted for comparison. Data for global subducting sediment (GLOSS) are from Plank and Langmuir^44^. Fields of UCC^42^, LCC^45^ and Indian Ocean MORB^46^ are obtained from the literature.

As seen in the chondrite-normalized REE patterns (Fig. 5C), all the granitoids in our study display nearly overlapping patterns with that of the UCC, indicating more enriched LREE compositions than for the LCC and the Indian Ocean sediments. In addition, both granitoids and volcanic rocks from our study have significant negative Eu anomalies, which are very similar to those of the UCC (average Eu/Eu* = 0.65^27^) and contrast with the positive Eu anomalies in the LCC (average Eu/Eu* = 1.14^27^). Although these negative Eu anomalies are generally interpreted as the residues of plagioclase feldspar, a more direct cause is assumed here that reflects the Eu-depleted primary magma because of the low degree of fractional crystallization. In the ^87^Sr/^86^Sr–^143^Nd/^144^Nd isotope space (Fig. 8), it is seen that compared with other magmatic rocks, including other Linzizong volcanic rocks, the Paleocene S-type magmatism shows an obvious increase in ^87^Sr/^86^Sr, confirming UCC affinity. The same conclusion also can be reached from the radiogenic Pb isotopes (Supplementary Table S3).

Two potential scenarios can be envisaged for formation of the Sinongduo S-type granitic rocks: (1) crustal anatexis^40,41^; (2) extraction from shallow andesitic magma reservoir by fractional crystallization^12^. For the crustal anatexis hypothesis, a deep buried metasedimentary source is required for the generation of S-type magma^47^. Exhumation of the overthickened crust is a widely accepted mechanism for the formation of strongly peraluminous S-type granites in post-collisional setting^34,47^. However, this model is inapplicable for the S-type granites formed non-collisional setting. A two-stage rollback of the subducted oceanic crust that caused crustal extension is proposed to account for the S-type granites in the Phanerozoic circum-Pacific orogenic belts^40^. S-type granites formed by this mechanism are consistently associated with high-temperature–low-pressure (HTLP) metamorphic complexes, or even some core complexes which were exhumed during continental extension^48,49^. A thinned lithosphere is also required for a substantial transient heat flux to the crust^40^. Overall, the Sinongduo S-type magmatic rocks cannot be correlated to both these models because HTLP metamorphic complex is absent and continental collision had not yet occurred at that time. Besides, the S-type magma cannot be extracted from shallow andesitic magma reservoir because of the low degree fractional crystallization. This hypothesis is also limited in explaining the large outcrop of these volcanic and intrusive rocks that cover more than 300 km^2^ in the study area (Fig. 2A,B).

As the Gangdese batholith and Linzizong volcanic rocks are treated as products of the northward subduction of the Neo-Tethys oceanic crust^6,9^, we propose an alternative scenario in which the Linzizong S-type volcanic rocks and granitoids are derived from subducted materials of UCC geochemical affinity. Zircon saturation temperatures (*T*zr) of the Sinongduo granitoids are in the range of 768–804 °C (mean = 793 °C) based on the method of Boehnke et al.^50^. The absence of inherited zircons in the granitoids indicates that partial melting took place at high-temperature conditions and the initial magmas were undersaturated in zirconium. In this case, the calculated zircon saturation temperatures provide minimum estimates of melting temperature^51^. Besides, microscopic observation indicates that the granitoids contain fewer zircon crystals. These features, together with the wide distribution of the volcanic rocks suggest that the melting temperature exceeded 804 °C, comparable to hot granites^51^. The substantial heat flux for melting can be achieved if these UCC subducted to a depth of lithospheric mantle.

## Implications for the India–Asia convergence

Our first assumption is that the subducted UCC represents a portion of the northern edge of the Indian plate, in which case the initial India–Asia collision started at approximately 65 Ma, which is in accordance with the popular geophysical collision model that shows continental lithosphere beneath southern Tibet^52,53^. However, it conflicts with most published studies that support collision ages to range from Late Cretaceous to Oligocene^54^, especially the recently accepted 55 ± 5 Ma age for the onset of collision^54–57^. On the other hand, Andean-type calc-alkaline magmatism with consistent isotopic compositions from the Early Jurassic to the middle Eocene^6,9^ naturally implies the persistence of Neo-Tethys oceanic crust subduction beyond a collisional background and suggests that the 65 Ma age for the India–Asia collision should be excluded^56^. Hence, the subducted UCC cannot be the northern edge of the Indian plate, and should be attributed to other continental subduction systems.

**Figure 9:**
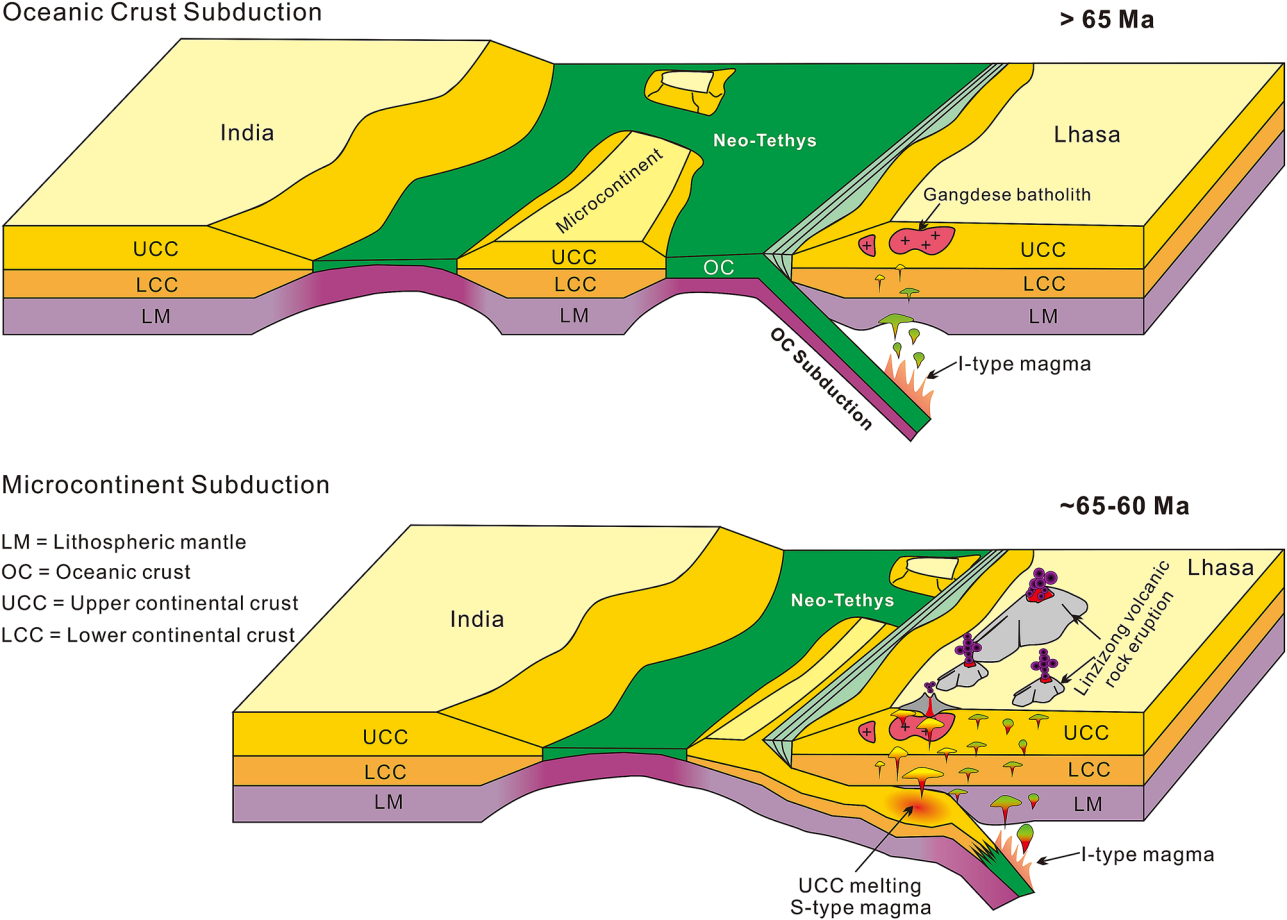
Conceptual model showing the subduction of the microcontinent and the formation of S-type magma. Not drawn to scale. See text for discussion.

An alternate possibility would be the subduction of microcontinents (microplates). Fragmentation and microcontinent formation are common phenomena in many continental margins^57^. The opening of the embryonic Neo-Tethys may have caused rifting of the northern margin of the Indian plate under a divergent tectonic setting and formed a cluster of microcontinents (Fig. 9) between the Indian plate and the Asia plate^58^. Besides, thermo-mechanical study reveals that the thermal and buoyancy effects of mantle plume impingement on the bottom of the continental part of a subducting plate can also induce the separation of microcontinents from the main body of the continent^59^. With the initiation of the northward subduction of the Neo-Tethys crust in early Mesozoic^60^, possibly induced by the impingement of mantle plume at the transition zone between oceanic and continental lithosphere^61^, these microcontinents are thought to have accreted to the Lhasa block along the IYSZ in the early Cenozoic, similar to the Burma terrane^62^ and the Oaxaquia in North America^63^. However, no geological records have been reported to document the accretion in the IYSZ.

In general, strong rheological coupling of UCC and LCC can separate the continental crust from the downgoing mantle lithosphere, while low rheological coupling of the UCC and LCC allows the LCC to sink into the mantle, resulting in continental subduction^64^. Numerical models indicate subduction of the Indian LCC^65^, which is also confirmed from geophysical evidence^52,53^. However, the possibility and mechanism of the subduction and recycling of the UCC remain poorly understood. Numerical modelling studies indicate that the UCC can subduct to a great depth if the continental crust is strongly coupled with the mantle lithosphere under a relatively low Moho temperature^66–68^. Another critical factor controlling the accretion and scale of continental subduction is the continental mass^69^, and compared with the short-subducted lengths of intact continental crust, microcontinents can be entirely subducted^70^. The coherence may play an important role in the subducting plate if the detachment did not occur^71^. A so called “crustal pocket” can subduct to a depth of 50–120 km^67^. Microcontinents might undergo subduction process comparable to that of the “crustal pocket” in the subduction zone. The melts derived from the subducted UCC are immediately transported to the surface across the rheologically coupled lithosphere^72^ along deep-seated lithospheric-scale faults that formed in the overriding plate^73^. We consider this mechanism for the S-type magmatic rocks that are distributed along the regional Luobadui-Milashan fault (LMF) as clusters (Fig. 2A). We thus propose that the microcontinents rifted from the India plate could have been wholly subducted without any accretion to the collision boundary.

If the whole UCC was subducted, then little or possibly no trace of accretion may be recorded in the suture zone, as these were entirely erased. Only the melting of UCC components can induce secondary geochemical consequences which are traceable from magmatic signatures^42^. After the subduction of the UCC, a significant period of oceanic crust subduction must have followed prior to the continental collision (Fig. 9). Our model has the advantage of reconciling the heterogeneity of the Linzizong volcanic rocks and coeval batholiths and can also support the subsequent India–Asia collision geodynamics.

Previous studies considered S-type magma to be a product of crustal anatexis^40^. However, our proposal opens up alternate possibilities of partial melting and recycling of subducted UCC, particularly involving microcontinents.

## Methods

### Zircon U–Pb dating

The granitoid samples for LA–ICP–MS zircon U–Pb dating were crushed, and zircons were separated using conventional heavy liquid and magnetic separation techniques. Zircons were handpicked under a microscope, mounted in epoxy resin, polished to approximately half their original thickness, and studied under both reflected and transmitted light. To examine the internal structure, cathodoluminescence (CL) images of zircon grains were obtained using a JSM6510 scanning electron microscope. Zircon U–Pb dating was performed using a Neptune MC-ICP-MS coupled with a New Wave UP213 laser ablation system at the Institute of Mineral Resources, Chinese Academy of Geological Sciences. The operating conditions and detailed analytical procedures followed those described by Hou et al.^74^. The U–Pb ages of the zircons were calculated and plotted using the Isoplot3 software (Ludwig, 2003). Individual analyses are presented with 1σ error, whereas age uncertainties are quoted at the 95% level (2σ).

### Whole-rock major and trace element analyses

Whole-rock major element analyses and trace element analyses were conducted with an X-ray fluorescence (XRF) spectrometer (Primus II, Rigaku, Japan) and an Agilent 7700e ICP-MS system, respectively, at the Wuhan Sample Solution Analytical Technology Co., Ltd., Wuhan, China. The sample powder was accurately weighed and mixed with the cosolvent (Li_2_B_4_O_7_: LiBO_2_: LiF = 9:2:1) and oxidant (NH_4_NO_3_) in a Pt crucible, which was then placed in the furnace at 1150 °C for 14 min. Next, the melted sample was saturated with air for 1 min to produce flat disks on the firebrick for the XRF analyses. Approximately 1 ml of HNO_3_ and 1 ml of HF were slowly added to a 50 mg sample of powder in a Teflon bomb, which was placed in a stainless-steel pressure jacket and heated to 190 °C in an oven for > 24 h. After evaporating the sample to dryness twice, 1 ml of HNO_3_, 1 ml of Milli-Q (MQ) water, and 1 ml of 1 ppm internal standard solution were added, and the Teflon bomb was resealed and placed in the oven at 190 °C for > 12 h. The final solution was transferred to a polyethylene bottle and diluted to 100 g by the addition of 2% HNO_3_ for ICP-MS analysis. Analyses of international rock standards (AGV-2, BHVO-2 and BCR-2) indicated that the precision and accuracy of the results were better than 5%.

### Whole-rock Sr–Nd–Pb isotope analyses

The Sr–Nd–Pb isotope ratios were determined by using a Finnigan Triton thermal ionization mass spectrometer (TIMS) at the State Key Laboratory for Mineral Deposits Research, Nanjing University. The measured ^87^Sr/^86^Sr and ^143^Nd/^144^Nd isotope ratios were normalized to ^86^Sr/^88^Sr = 0.1194 and ^146^Nd/^144^Nd = 0.7219, respectively, for mass fractionation correction. During the period of data acquisition, the mean ^87^Sr/^86^Sr ratio of the Sr standard (NBS987) and the ^143^Nd/^144^Nd ratio of the Nd standard (JNDi-1) were 0.710261 ± 0.00006 and 0.512128 ± 0.00004 (2σ), respectively. Further, the measured ^87^Sr/^86^Sr and ^143^Nd/^144^Nd values for the standard BCR-2 were 0.705018 ± 0.000003 and 0.512624 ± 0.000004, respectively (the equivalent reference values obtained from Weis et al.^75^ are 0.705019 ± 0.000016 and 0.512634 ± 0.000012). The analytical procedures for Sr and Nd isotopes followed those described in detail by Pu et al.^76^. The value of *ε*_Nd_(t) was calculated with reference to the chondritic uniform reservoir (CHUR) ^143^Nd/^144^Nd ratio of 0.512638.

For Pb isotope analysis, sample powders were weighed into a Teflon bomb and dissolved by a combination of purified HNO_3_ and HF at 190 °C for 48 h. Pb was separated and purified on ion exchange columns with diluted HBr as the eluant. Finally, the Pb fraction was eluted using 6.0 M HCl and gently evaporated to dryness prior to mass spectrometric measurement. The detailed procedures used in measuring Pb isotopes can be found in White et al.^77^. The measured Pb isotopic ratios were rectified for instrumental mass fractionation by performing replicate analyses of the standard NIST-981. The standard NIST-981 yielded ^206^Pb/^204^Pb = 16.9318 ± 0.0003, ^207^Pb/^204^Pb = 15.4858 ± 0.0003, and ^208^Pb/^204^Pb = 36.6819 ± 0.0008. In addition, the international standard BCR-2 was verified using an unknown sample by employing this method. The measured values for the BCR-2 Pb standard were 18.7881 ± 0.0005 for ^206^Pb/^204^Pb, 15.6196 ± 0.0004 for ^207^Pb/^204^Pb, and 38.8005 ± 0.0010 for ^208^Pb/^204^Pb (reference values can be found in Weis et al.^75^).

## Supplementary Information

Supplementary Table S1.

Supplementary Table S2.

Supplementary Table S3.

## Data Availability

All data generated or analysed during this study are included in this published article (and its Supplementary Information files).
